# Brain parcellation for TMD neuroimaging: a critical narrative review

**DOI:** 10.1038/s41405-026-00407-2

**Published:** 2026-04-21

**Authors:** Natalia Savychuk, Vasyl Pekhno, Anastasiia Liakhovska, Roman Sulik, Ivan Riabko

**Affiliations:** 1 Department of Therapeutic and Pediatric Dentistry, Shupyk National Healthcare University of Ukraine, Kyiv, Ukraine; 2 Department of Therapeutic and Pediatric Dentistry, Department of Orthopedic Dentistry, Digital Technologies and Implantology, Shupyk National Healthcare University of Ukraine, Kyiv, Ukraine; 3 Department of Orthodontics, Poltava State Medical University, Poltava, Ukraine; 4 Department of Neurology, Shupyk National Healthcare University of Ukraine, Kyiv, Ukraine; 5 Department of Medical Physics, Taras Shevchenko National University of Kyiv, Kyiv, Ukraine

**Keywords:** Malocclusion, Mandibular muscles, Orofacial pain

## Abstract

**Purpose:**

To analyze modern brain mapping for neurobiological mechanisms of temporomandibular disorders, with particular emphasis on structural and functional alterations, employing advanced neuroimaging techniques such as fMRI and DTI. Furthermore, this study aims to identify the most appropriate combination of brain parcellation schemes that comprehensively cover cortical, subcortical, and brainstem structures to enhance the accuracy and standardization of neuroimaging protocols in TMD research.

**Methods:**

Scientific sources were searched in PubMed, Scopus, Web of Science and Google Scholar as of 29.06.2025, 689 records were identified for PRISMA workflow, of which 676 records remained for screening after the removing duplicates and irrelevant items, 630 records were excluded per prespecified criteria (inappropriate diagnoses, insufficiently described rapid studies, metabolic disorders), and 46 studies were included in the qualitative analysis.

**Results:**

We compare anatomical, functional, and multimodal atlases (Desikan–Killiany, Destrieux, Schaefer, HCP-MMP1.0, Brainnetome, SUIT, Brainstem atlases) in terms of fMRI/DTI compatibility and relevance of regions of interest.

**Conclusions:**

The authors suggest that the use of a combined parcellation scheme: HCP-MMP1.0 for the highly detailed cortex, SUIT for the cerebellum, specialized brainstem atlases—which cover corticocortical, cerebellar, and brainstem connections—increases mapping accuracy and meets modern requirements for the standardization of neuroimaging protocols in studies of the TMD.

## Introduction

Temporomandibular disorders (TMD)—heterogeneous group of musculoskeletal and neuropathic conditions characterized by chronic orofacial pain, restricted jaw mobility, and joint dysfunction. Although the relationship with pain severity remains inconsistent, in many patients, no clear peripheral pathology is sufficient to explain symptom persistence. This discrepancy points to a significant contribution of central nervous system (CNS) mechanisms to the development and maintenance of chronic TMD pain. Over the past decade, functional MRI (fMRI) and diffusion tensor imaging (DTI)—has become central to studying the neurobiological basis of TMD. These modalities consistently demonstrate structural and functional alterations within cortical and subcortical systems responsible for sensory–discriminative processing, motor control, affective modulation, and descending antinociception. The validity of such findings depends heavily on accurate brain parcellation, which defines regions of interest (ROIs) for quantitative assessment. Parcellation schemes—anatomical, functional, or multimodal—must be selected according to the research aims [1–6]. The TMD involves distributed circuits encompassing sensorimotor, salience, limbic, and default-mode networks, as well as cerebellar and brainstem centers implicated in pain modulation. Therefore, combining cortical atlases, cerebellar parcellation, and brainstem atlases can provide a more complete and reproducible framework [7, 8]. Morphometric and functional studies reveal associations between cortical thickness, gray-matter density, pain sensitivity, and altered activation of the insula, cingulate, thalamus, and basal ganglia [9–12]. A 2020 systematic review summarized distributed abnormalities across the trigeminal nuclei, thalamus, insula, anterior cingulate cortex (ACC), sensorimotor cortices, periaqueductal gray, and prefrontal regions1. More recent studies highlight widespread network-level dysfunction affecting the pain matrix, salience network, and default mode network [13–16].

In our opinion, these converging findings underscore the need for standardized neuroimaging protocols to improve reproducibility, harmonize ROIs, and facilitate integration of multimodal evidence. A unified parcellation strategy that consistently represents cortical, cerebellar, and brainstem structures is essential for capturing the distributed neurobiological alterations characteristic of TMD and advancing mechanistic understanding that can inform future clinical interventions.

## Methods

### Search strategy

We systematically searched PubMed, Scopus, Web of Science, and Google Scholar (June 29, 2025) using combinations of “Neuroimaging,” “MRI,” “fMRI,” “Temporomandibular disorders (TMD),” and “Parcellation atlases,” with AND/OR and MeSH (PubMed). No language or year limits were applied.

#### Eligibility criteria

Of 689 records, 13 duplicates were removed (*n* = 676 screened by titles/abstracts). We included original studies, clinical cases, and meta-analyses using neuroimaging (fMRI, DTI) and brain parcellation in TMD. We excluded duplicates, items without full text, studies unrelated to TMD, rapid studies lacking methods, and works on metabolic disorders.

#### Data extraction and quality assessment

Full texts were retrieved when available and assessed against the criteria without restrictions on language, year, or population.

#### Data synthesis

**Fig. 1:**
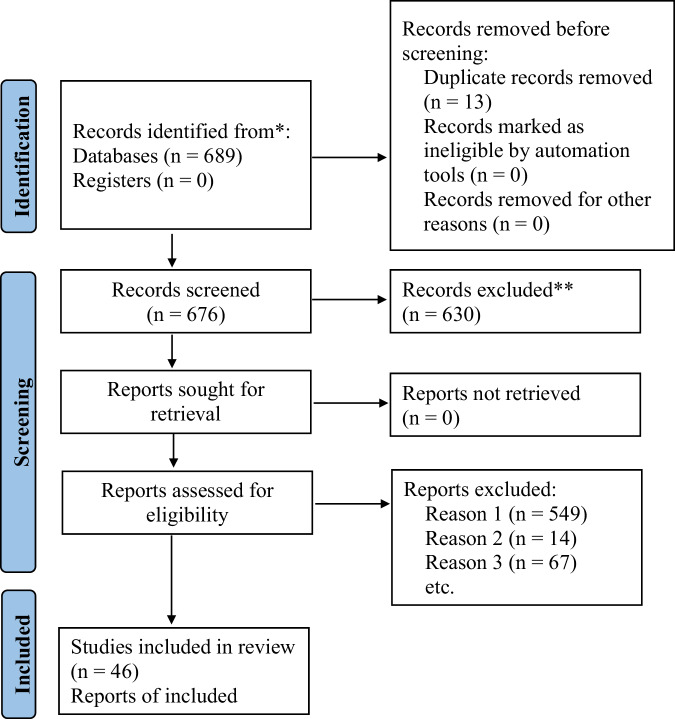
PRISMA flow diagram of the literature search and study selection. Boxes summarize the numbers of records identified, screened, assessed for eligibility, and included in the qualitative synthesis; arrows indicate the screening pathway, and n denotes the number of records, reports, or studies at each stage.

After exclusions—neurological disorders not directly related to TMD (*n* = 584), insufficiently described rapid studies (*n* = 14), and metabolic disorders (*n* = 67)—24 unique studies (24 full-text reports) were included in the qualitative synthesis (Fig. 1).

## Results

### Neuroimaging methods and brain parcellation schemes

Neuroimaging methods such as fMRI measure blood oxygenation level–dependent (BOLD) fluctuations and are the principal tool for identifying functional network reorganization across cortical, cerebellar, and brainstem regions. Both task-based and resting-state paradigms are applied: task fMRI isolates stimulus- or motor-related responses, whereas resting-state fMRI characterizes intrinsic connectivity within sensorimotor, salience, and pain-modulatory networks [17–19]. DTI complements fMRI by mapping white-matter pathways, including trigeminothalamic, corticopontine, cerebello-thalamic, and descending antinociceptive tracts. VBM provides voxel-wise quantification of gray-matter density and cortical thickness, while MRS measures neurochemical markers and excitatory–inhibitory balance (e.g., glutamate/GABA ratios), contributing to a multidimensional characterization of neuroplastic changes in TMD [17]. More than 60 human brain atlases have been defined by distinct criteria, such as cytoarchitecture, functional coactivation, connectivity, or multimodal integration [20]. Consequently, atlas selection must be hypothesis-driven and aligned with the imaging modality, spatial scale, and target neural system. No single parcellation scheme is universally optimal. Cortical atlases vary in anatomical, cytoarchitectonic, and functional definitions and therefore differ in suitability depending on the research question and data resolution [21–26].

#### Clinical and neurobiological specificity

Parcellation should explicitly include regions implicated in TMD pain and motor control, such as the primary and secondary sensorimotor cortices, insula, amygdala, thalamus, basal ganglia, cerebellar lobules and nuclei, and descending antinociceptive structures like the periaqueductal gray (PAG) and rostral ventromedial medulla (RVM). The parcellation must be granular enough to separate adjacent yet functionally distinct territories (e.g., anterior vs. posterior insula, orofacial S1 vs. M1 representations).

#### Functional vs. anatomical priority (fit to the research question)

If the aim is network-level inference (e.g., default mode, salience, or somatomotor systems), functionally derived parcellations are preferable because they capture coherent time-series structure and canonical network organization. Conversely, for precise anatomical localization or structure–function mapping, anatomically or cytoarchitectonically defined atlases—particularly those resolving gyri, sulci, or brainstem nuclei—should be prioritized.

#### Modality and pipeline compatibility

The parcellation must align with the data modality and preprocessing pipeline. For fMRI, consider differences between task-based and resting-state paradigms, including HRF modeling, temporal filtering, and smoothing. For DTI, select atlases consistent with the angular and spatial resolution of the diffusion data and tractography method. Ensure that atlases are available in the same stereotaxic space as the workflow, e.g., Montreal Neurological Institute (MNI), and represent the average anatomy of 152 healthy adult subjects. (MNI152), FreeSurfer–Leftright (fsLR) and those mappings are reported explicitly.

#### Prior use and comparability

Selecting atlases widely employed in pain and TMD neuroimaging enhances reproducibility and enables meta-analytic synthesis. When novel or less common atlases are adopted—such as those resolving specific brainstem nuclei—authors should provide clear justification, note prior validation studies, and, when feasible, include a mapping to canonical parcellations to maintain cross-study comparability [3, 21–26].

### Practicality and reproducibility

Prefer openly available, versioned resources with transparent licensing and stable ROI identifiers. Atlases should have broad compatibility across major software packages (FSL, SPM, AFNI, FreeSurfer, Connectome Workbench). A predefined ROI inclusion list, minimal voxel-count thresholds, and explicit quality-control criteria should be provided to ensure replicability of the analysis.

#### Implications for fMRI/DTI interpretation

To interpret fMRI signals and diffusion metrics in a TMD context, the chosen parcellation must:

1. Cover all relevant systems (sensorimotor, salience/limbic, thalamic/basal ganglia nodes, cerebellum, trigeminal complex, and descending antinociceptive structures) at a resolution adequate to separate adjacent but functionally distinct territories;
2. Be compatible with acquisition and preprocessing, i.e., voxel size, spatial smoothing, TR/temporal filtering (for fMRI), and angular/spatial resolution and tractography approach (for diffusion MRI);
3. Maintain spatial correspondence across structures (cortex–cerebellum–brainstem) in a common stereotaxic space to enable integrated structural–functional analyses and minimize partial-volume and misregistration effects.

### Selecting a parcellation scheme for TMD studies (authors hypothesis)

#### Specificity to TMD-relevant systems

Ensure coverage of ROIs in TMD pain and motor control: primary/secondary sensorimotor cortices, insula, amygdala, thalamus, basal ganglia, cerebellar lobules/nuclei, trigeminal complex, and descending antinociceptive structures (PAG, RVM). The chosen parcellation should be granular enough to separate functionally distinct but anatomically adjacent areas. Functional–anatomical balance (fit to the research question): if the primary aim is to study network-level interactions**—**such as connectivity within the default mode network, salience, or somatomotor systems—use functionally derived parcellations that capture coherent temporal activity patterns. If the goal is precise anatomical localization or structure–function correlation, prefer anatomical or cytoarchitectonic atlases. For multimodal integration (fMRI–DTI), using multimodal atlases that align cortical, subcortical, and cerebellar representations.

Anatomical cortical schemes such as Desikan–Killiany provide gyral-based parcellation with moderate granularity suitable for stable ROI-level analyses, whereas the Destrieux atlas refines these boundaries by distinguishing both gyri and sulci, improving localization at the cost of lower SNR and more complex alignment requirements. Multimodal approaches such as HCP-MMP1.0 delineate 360 cortical areas from combined structural, functional, and myelin information, enabling high-resolution network analyses. Functionally derived atlases such as Schaefer offer 100–1000 parcels aligned to the Yeo 7/17-network model, supporting flexible granularity for resting-state and task-based fMRI. For infratentorial structures, the SUIT atlas ensures accurate cerebellar normalization and lobular parcellation, while specialized brainstem atlases provide nucleus-level ROIs for key nociceptive and antinociceptive pathways (e.g., trigeminal complex, PAG, RVM). Connectivity-oriented resources such as the Brainnetome atlas integrate cortical, subcortical, and cerebellar regions based on multimodal profiles, supporting structure–function analyses. The Gordon atlas defines 333 resting-state parcels for network-centric investigations, whereas the Talairach coordinate system remains relevant primarily for spatial reference and legacy data. Consensus-based resources such as CAREN and MICCAI datasets serve as benchmarks for network alignment and segmentation validation rather than direct ROI parcellation. The Archer Sensorimotor Tract Template provides tract-based masks for descending corticofugal pathways and is useful for DTI studies of sensorimotor and pain-related motor modulation. The more detailed information in the text below.

**Fig. 2:**
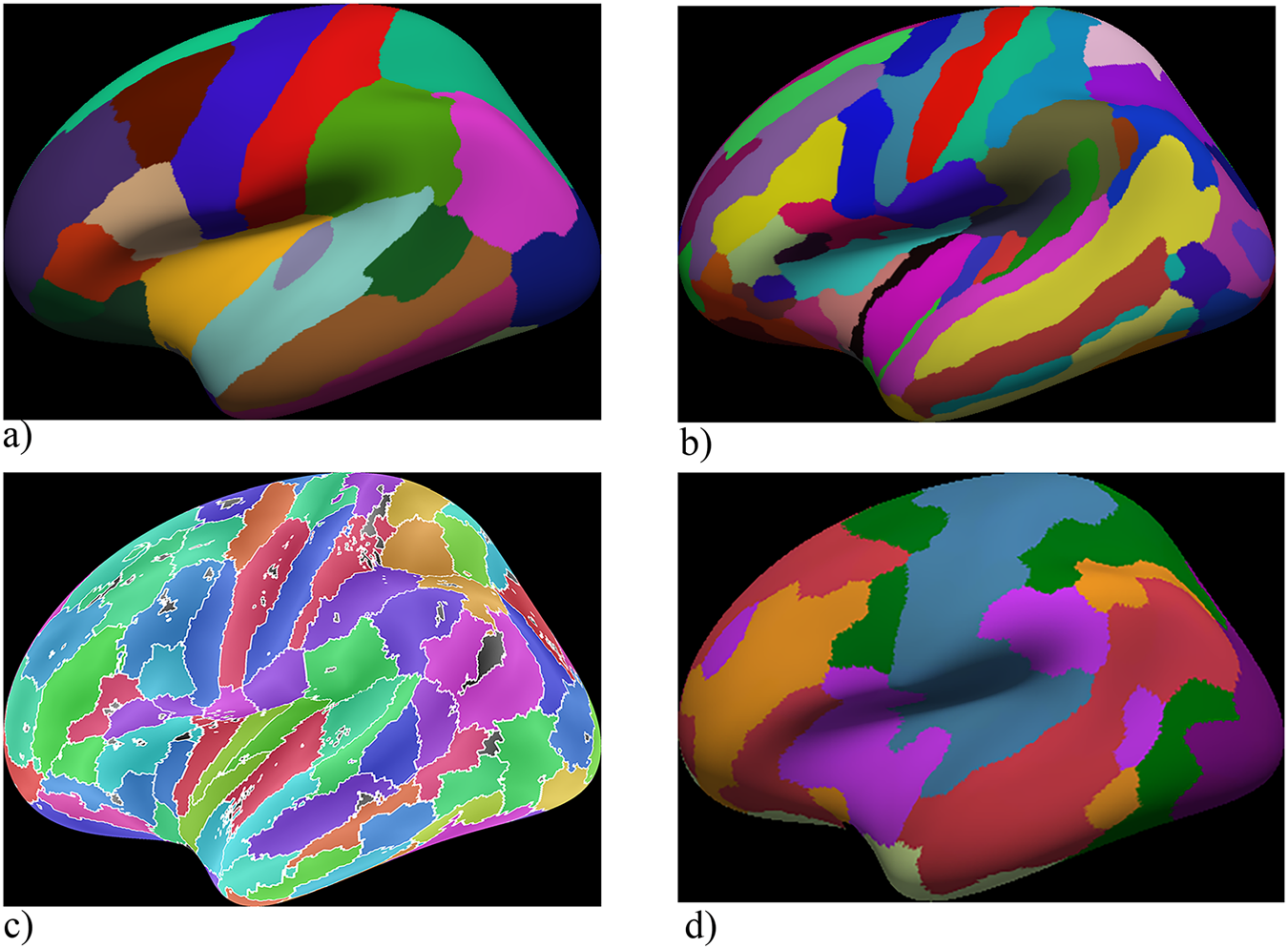
Representative cortical parcellation atlases. **a** Desikan–Killiany atlas; **b** Destrieux atlas; **c** HCP-MMP1.0 atlas; **d** Schaefer atlas. Different colors indicate distinct cortical parcels/regions of interest, and boundary lines delineate parcel borders on representative surface renderings.

Desikan–Killiany (DK; Fig. 2a) is a widely used gyral-based cortical parcellation comprising 68 regions (34 per hemisphere), routinely implemented in standard pipelines; its labels can be mapped from the cortical surface to volumetric space for ROI-based analyses. Strengths: DK provides anatomically interpretable and stable gyral/sulcal boundaries, facilitating reporting and cross-study comparability. Its moderate granularity yields robust ROI-level signal-to-noise characteristics, supporting reliable extraction of BOLD signals, cortical thickness, and curvature metrics, as well as construction of 68 × 68 connectivity matrices. Limitations: DK does not include subcortical structures, cerebellum, or brainstem, which require complementary resources. Its relatively coarse functional specificity means that large parcels (e.g., precentral/postcentral gyri, insula, cingulate) may pool heterogeneous functional territories, increasing partial-volume effects and potentially obscuring TMD-related focal alterations. Parcel-size variability may interact with smoothing and motion-correction procedures; reporting ROI voxel counts and testing sensitivity to smoothing are recommended. Consistent stereotaxic space (MNI or fsLR) must be ensured when integrating DK with diffusion-based analyses. Practical guidance for TMD studies: DK is useful as a baseline anatomical framework for whole-cortex summaries and moderate-scale connectivity analyses. For hypotheses involving small or functionally specialized regions (e.g., orofacial S1/M1, anterior vs. posterior insula), DK should be complemented by higher-resolution atlases for complete cortical–cerebellar–brainstem coverage. Spatial smoothing should be conservative relative to the smallest DK parcels to minimize signal leakage. Updates: refinements such as the Mindboggle/FreeSurfer-compatible DK variant improve anatomical consistency and inter-subject homology while retaining similar granularity, and remain widely used in contemporary pain and cognitive-control research [27–30].

Destrieux (Fig. 2b)—cortical anatomical parcellation with 148 regions total (74 per each hemisphere) that encodes both gyri and sulci, yielding higher fidelity to cortical geometry than DK. Labels are defined on the surface and can be mapped to volume for ROI analyses. Strengths: (1) Greater localization granularity for fMRI/DTI than DK (e.g., separates anterior vs. posterior insula; subdivides cingulate and perirolandic regions), which can better isolate orofacial sensorimotor territories and pain-relevant subdivisions; (2) Anatomically principled boundaries improve interpretability for structure–function claims and tract-anchored analyses. Scope and limitations: (1) Cortex-only: no detailed segmentation of subcortex, cerebellum, or brainstem; pair with FreeSurfer aseg/subcortical atlases, SUIT for cerebellum, and dedicated brainstem nuclei atlases; (2) SNR/partial-volume trade-off: finer parcels can reduce ROI-level SNR and increase sensitivity to misregistration and smoothing leakage; parcel-size heterogeneity also increases variance across ROIs; (3) Registration complexity: sulcal variability across subjects can complicate cross-subject alignment; report surfaces/space and QC. Possible practical guidance for TMD study could be when hypotheses require finer anatomical differentiation within the insula, cingulate, and perirolandic face areas [31].

Human Connectome Project Multi-Modal Parcellation (HCP-MMP1.0; Fig. 2c) is a multimodal cortical atlas with 360 areas (180 per hemisphere) defined by converging evidence from cortical myelin (T1w/T2w), thickness, task activations, resting-state connectivity, and topographic features [3, 32, 33]. Strengths: high areal specificity and reproducibility: suitable for detecting subtle effects in insula subfields, SMA/pre-SMA, premotor subdivisions, and prefrontal territories; network fidelity: parcels respect canonical functional networks while preserving fine spatial differentiation, enabling precise ROI-level and connectomic analyses; mature tooling: full support in Connectome Workbench/HCP pipelines; straightforward extraction of parcel-wise BOLD time series and construction of high-resolution connectivity matrices. Limitations: cortex-only; surface dependence: mapping HCP-MMP1.0 to group volume space degrades areal boundaries (blurring/partial volume); parcel size vs. SNR: fine parcels can lower ROI-level SNR and increase sensitivity to misregistration and smoothing; report parcel sizes and consider conservative smoothing. Possible practical guidance: use HCP-MMP1.0 when hypotheses require fine-grained cortical localization; keep spatial smoothing modest (e.g., ≤4–6 mm FWHM on the surface) and verify surface-to-volume mappings if volumetric statistics are required; harmonize spaces across modalities (fMRI/DTI) and combine with another atlases [3, 32, 33].

Schaefer (local–global; Fig. 2d)—Functional parcellation comprising 100–1000 regions per hemisphere, aligned with the Yeo 7- and 17-network frameworks, approach integrates local gradients of functional connectivity with global network topology, offering a flexible balance between spatial resolution and network coherence [3, 34, 35]. Strengths: provides flexible granularity (commonly 200–400 or 600 ROIs); preserves canonical resting-state networks—including somatomotor, salience, and default mode systems; alignment with the Yeo network model enhances cross-study comparability with existing pain, cognitive-control, and attention research. Limitations: does not include subcortical, cerebellar, or brainstem regions; higher-resolution variants reduce ROI-level SNR and increase vulnerability to motion, misregistration, and smoothing effects. Volumetric projection of surface-defined parcels can blur sulcal boundaries; surface-based analysis is preferred. Practical guidance for TMD studies: Intermediate resolutions (200–400 ROIs) offer a favorable balance between localization precision and signal stability and are particularly effective for resolving orofacial sensorimotor, insular, and anterior cingulate regions implicated in TMD. For comprehensive assessment of TMD-related mechanisms, Schaefer should be combined with another atlases to ensure continuous coverage across the cortex–cerebellum–brainstem axis [3, 34, 35].

**Fig. 3:**
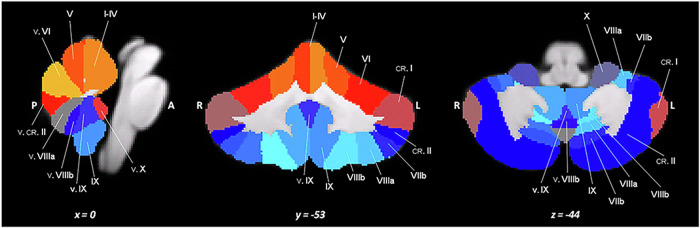
SUIT cerebellar parcellation atlas. From left to right, representative sagittal (*x* = 0), coronal (*y* = −53), and axial (*z* = −44) views are shown. Different colors indicate individual cerebellar lobules/parcels; labels denote lobular names; and L, R, A, and P indicate anatomical orientation (left, right, anterior, and posterior).

SUIT (Fig. 3): Spatially Unbiased Infratentorial Template atlas provides high-resolution cerebellar parcellation, covering lobules I–X, the vermis, and the deep cerebellar nuclei. Compared with whole-brain MNI normalization, SUIT offers superior anatomical alignment of infratentorial structures, minimizing folial deformation and improving localization of functional and structural findings [36, 37]. Strengths: SUIT delivers markedly improved registration accuracy for cerebellar fMRI and diffusion analyses, preserving native cerebellar morphology; supports ROI-based analyses of motor control, sensorimotor integration, cognitive functions, and pain modulation; suitable for multimodal work, including fMRI ROI extraction, tract-anchored DTI (e.g., cerebellar peduncles), and cerebello-cortical connectivity mapping. Limitations: excludes cortical, subcortical, and brainstem regions, which must be supplied by complementary resources; small lobular ROIs are susceptible to partial-volume and smoothing effects; thus, voxel size and preprocessing steps require careful optimization; accurate application depends on SUIT-specific normalization pipelines and rigorous quality control. Practical guidance for TMD studies: SUIT is recommended when investigating cerebellar, including pain modulation, sensorimotor adaptation, and movement-related cognitive control; minimal spatial smoothing (≤4 mm) and explicit reporting of ROI sizes and exclusions are advised; for complete coverage of TMD-relevant pathways, SUIT analyses should be integrated with another cortical parcellation and specialized brainstem atlases to achieve continuous representation of the cortex–cerebellum–brainstem axis [36, 37].

**Fig. 4:**
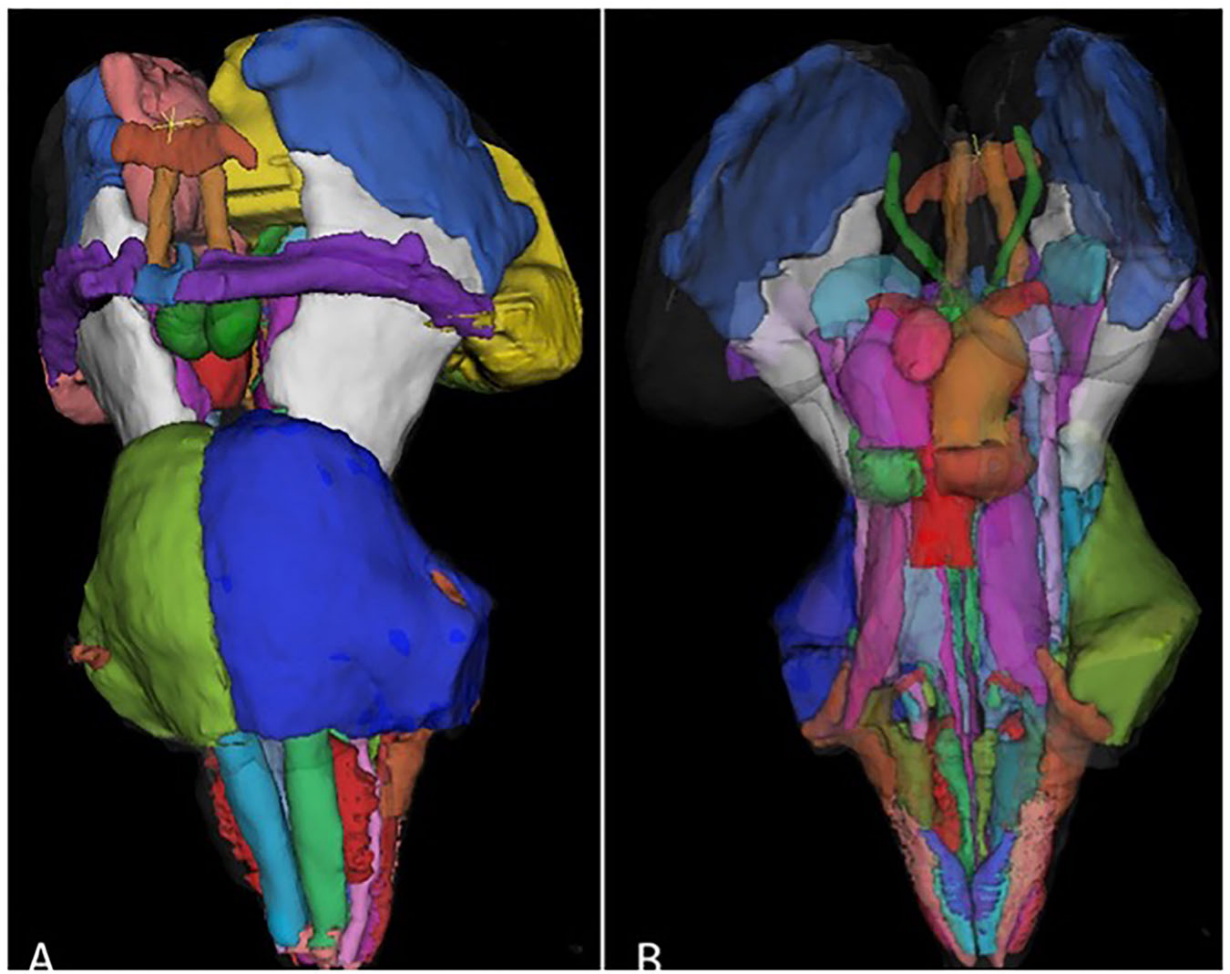
Brainstem atlas renderings. **a** Anterior surface view. **b** Posterior surface view with reduced opacity and with the thalamus and fourth ventricle removed to improve visualization of underlying structures. Different colors indicate distinct brainstem nuclei/regions of interest.

Brainstem atlases (Fig. 4): a heterogeneous family of regional and nucleus-level parcellations that vary in granularity, ranging from broad segmentation of the midbrain, pons, and medulla to detailed delineation of key nuclei such as the periaqueductal gray (PAG), rostral ventromedial medulla (RVM), locus coeruleus, raphe nuclei, nucleus tractus solitarius (NTS), and inferior olives. These resources capture core components of descending antinociceptive, autonomic, and arousal pathways that are central to pain modulation in TMD [38, 39]. Strengths: provide high neuroanatomical specificity, enabling precise localization and hypothesis-driven ROI analyses of nuclei involved in trigeminal nociception and descending inhibition (e.g., PAG–RVM axis) support multimodal integration, including fMRI studies of pain and autonomic regulation and DTI-based tractography of reticulospinal, spinoreticular, and cerebello–brainstem pathways. Limitations: requiring submillimeter spatial accuracy and, ideally, high-field MRI or advanced motion- and pulsation-suppression strategies. Standard MNI normalization may not preserve fine nuclei boundaries; therefore, brainstem-specific templates and careful manual quality control are essential. Parcellations differ substantially across atlases in boundaries, nomenclature, and reference space, making precise reporting of atlas name, version, and coordinate framework critical for reproducibility. Practical guidance for TMD studies: should be used when examining descending antinociceptive systems or trigeminal–autonomic interactions relevant to TMD; conservative smoothing ( ≤ 3 mm), physiological noise correction, and nucleus-level alignment checks are recommended.

For whole-axis coverage, brainstem ROIs should be combined with cortical atlases for cortical analyses, enabling integrated assessment of structural and functional connectivity across the cortex–cerebellum–brainstem axis [38, 39].

**Fig. 5:**
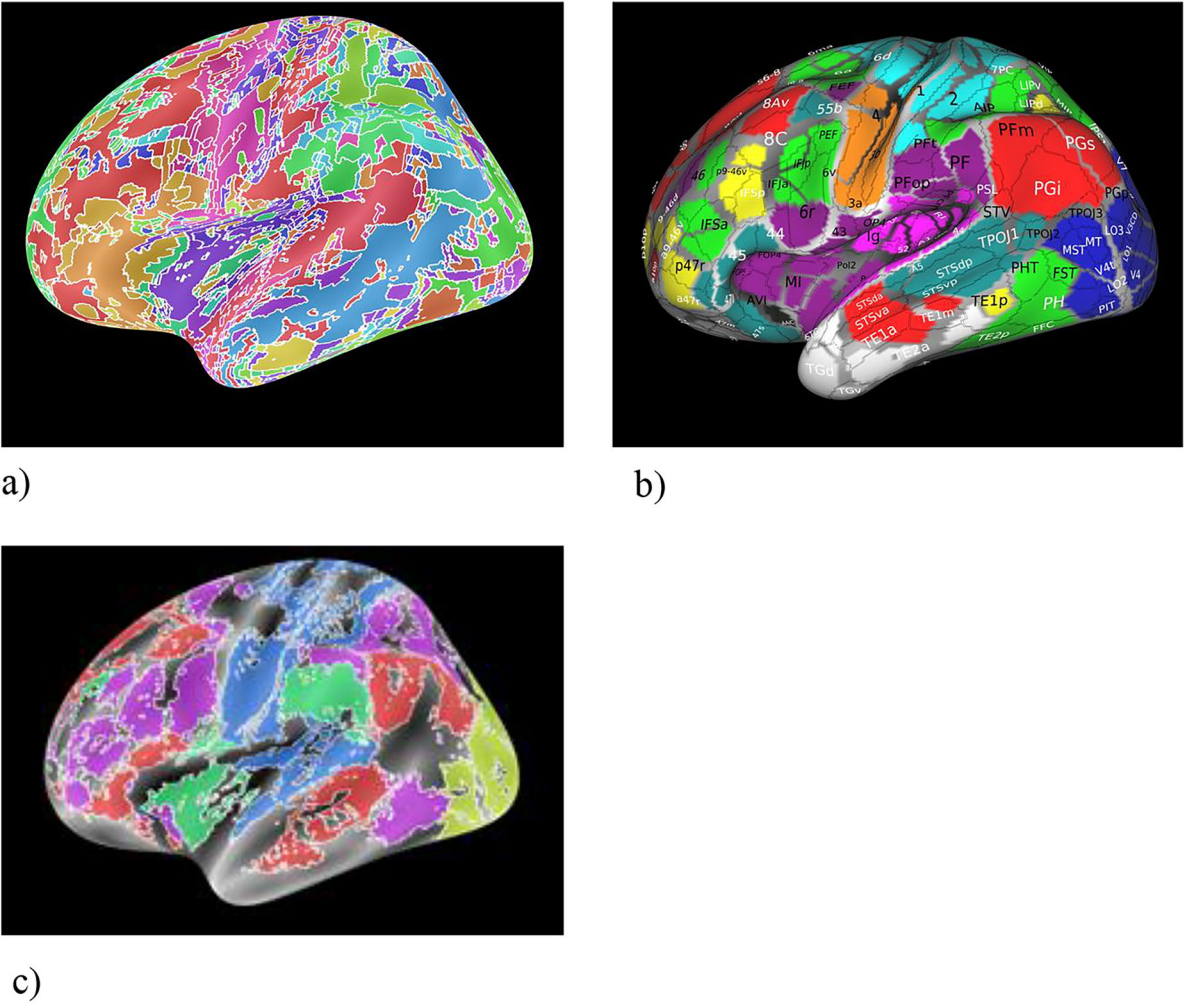
Reference and functional atlas examples relevant to TMD neuroimaging. **a** Talairach atlas; **b** Gordon atlas; **c** CAREN atlas. Different colors indicate distinct labeled cortical parcels or regions used for spatial reference, network definition, or segmentation benchmarking.

Talairach (Fig. 5a): one of the earliest atlases, without providing fine regional parcellation. Based on a single post-mortem brain, it established the foundation for early neuroimaging localization and remains a reference framework for reporting stereotaxic coordinates [40]. Strengths: facilitates interpretation of legacy neuroimaging literature that reported coordinates exclusively in this space; supports approximate ROI localization in older datasets and coordinate transformations to MNI space. Limitations: lacks of detailed parcellation; unsuitable for modern ROI-based or connectivity analyses; introduces anatomical bias and structural distortion when applied to population-level or high-resolution data; contemporary neuroimaging tools primarily rely on MNI152 or fsLR templates, limiting integration of Talairach coordinates into modern workflows. Practical guidance for TMD studies: can be used only as a spatial reference for interpreting or converting coordinates from earlier pain or TMD studies [40].

Gordon 2016 (Fig. 5b)—functional cortical parcellation comprising 333 regions derived from clustering surface-based resting-state fMRI connectivity patterns. The atlas provides high-resolution cortical coverage but excludes subcortical, cerebellar, and brainstem structures [41]. Strengths: closely with canonical resting-state systems, facilitating functional connectivity analyses, graph-theoretical metrics, and assessments of network modularity; parcels show strong within-region homogeneity and clear separation between networks; supports precise ROI extraction and cross-study comparability in cortical network research. Limitations: requires complementary parcellations (SUIT, or brainstem atlases, etc.) for whole-brain studies; parcel sizes vary across networks, which may influence SNR and statistical weighting; surface-based processing is recommended, as volumetric projection introduces alignment errors and partial-volume effects, especially around deep sulci. Practical guidance for TMD studies: useful for investigating large-scale functional connectivity and network-level alterations in TMD; particularly within somatomotor, salience, and default-mode systems; to achieve full-axis coverage from cortex to medulla, the Gordon atlas should be combined with SUIT for cerebellar parcellation and dedicated brainstem atlases [41].

Consensual Atlas of Resting-state Networks (CAREN) (Fig. 5c)—consensus parcellation of major cortical resting-state networks—such as the default mode, central executive, and attention systems—derived by integrating several widely used functional atlases. It is intended for harmonizing network-level analyses across studies rather than for detailed morphometry or subcortical, cerebellar, or brainstem segmentation [42]. Strengths: enables cross-atlas harmonization by reconciling labels from resting-state frameworks, supporting consistent network identification across datasets; well-suited for examining large-scale functional organization and inter-network connectivity; available in common stereotaxic spaces (MNI, fsaverage); easily integrated into group-level fMRI workflows. Limitations: excludes subcortical, cerebellar, and brainstem structures; limits fine-grained anatomical localization and ROI-based analyses; not optimized for DTI, structural morphometry, or nuclei-level investigations, restricting its applicability to network-level fMRI studies. Practical guidance for TMD studies: appropriate for investigating large-scale functional interactions relevant to TMD, such as altered connectivity between salience and default-mode systems or disruptions in top-down attentional modulation; cannot be used for DTI or detailed infratentorial analyses; should be paired with higher-resolution cortical atlases (e.g., HCP-MMP1.0 or Schaefer) complemented by SUIT and brainstem parcellations to achieve comprehensive coverage across the cortex–cerebellum–brainstem axis [42].

Medical Image Computing and Computer-Assisted Intervention (MICCAI 2012)—is not a functional ROI atlas but a reference collection of 30 manually labeled brain MRIs created. It serves primarily as a benchmark for evaluating segmentation algorithms and for developing or validating atlas-based methods, rather than as a parcellation scheme for fMRI or DTI studies [43]. Strengths: provides high-quality manual anatomical labels that serve as ground truth for assessing the accuracy and reproducibility of structural segmentation methods; widely used in benchmarking, algorithmic comparison, multi-atlas fusion pipelines, and machine-learning-based segmentation research. Limitations: not a functional or connectivity-based atlas and is therefore unsuitable for ROI definition in fMRI or diffusion analyses; small sample size (*n* = 30) limits generalizability for population-level inference; it does not provide practical cortical, cerebellar, or brainstem parcellations for neurofunctional studies. Practical guidance for TMD studies: should be used only for validating structural segmentation accuracy or for developing custom parcellation workflows; not appropriate for defining ROIs in functional or diffusion studies; TMD research should instead rely on anatomically or functionally grounded atlases—such as HCP-MMP1.0, Schaefer, SUIT, or dedicated brainstem parcellations—to ensure biologically meaningful and reproducible localization [43].

**Fig. 6:**
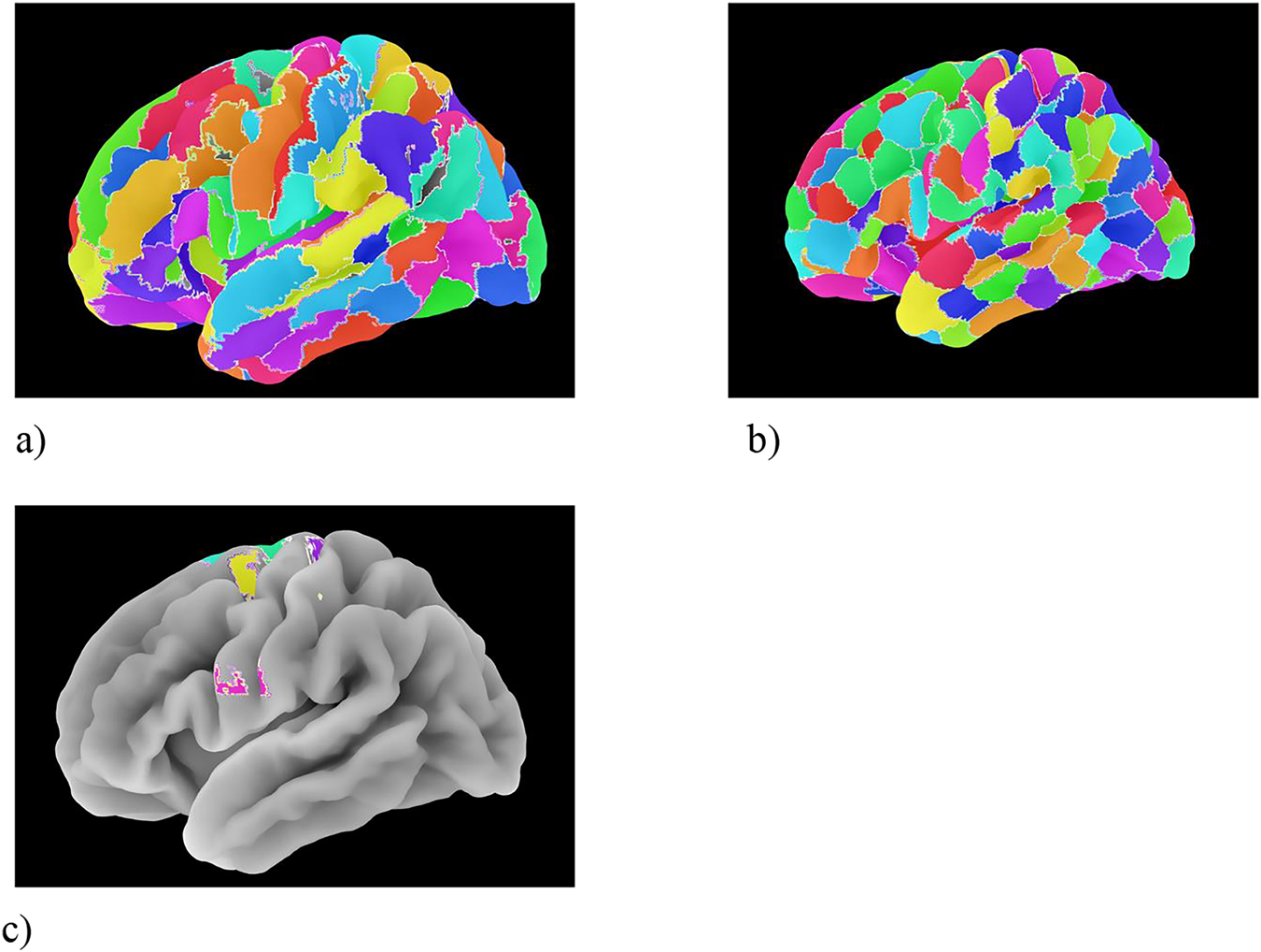
Multimodal and tract-based parcellation resources. **a** Brainnetome atlas; **b** Schaefer local–global parcellation; **c** Archer sensorimotor area tract template. Different colors indicate distinct cortical parcels in (**a**, **b**) and different probabilistic sensorimotor tract territories in (**c**).

Brainnetome (Fig. 6a)—a multimodal, connectivity-defined parcellation that subdivides the brain into 210 cortical, 36 subcortical, and 28 cerebellar regions based on combined diffusion MRI tractography and resting-state fMRI connectivity profiles. Each parcel reflects a connectivity-derived subregion with distinct structural and functional characteristics, accompanied by a comprehensive cross-modal connection matrix [3]. Strengths: integrates structural and functional connectivity, providing biologically grounded regional boundaries; broad coverage across cortical, subcortical, and cerebellar territories enables large-scale integrative analyses of brain networks; precomputed connectivity profiles support investigations of structural–functional coupling and graph-theoretical modeling. Limitations: does not include detailed brainstem parcellation, limiting resolution of small nuclei within the medulla, pons, and midbrain; complementary brainstem atlases are required for these structures; high parcel count and cross-modal data increase computational demands and require careful alignment across modalities; Spatial granularity is moderate—finer than classical anatomical atlases but coarser than high-resolution cortical schemes such as HCP-MMP1.0 (for example). Practical guidance for TMD studies: useful for examining network-level reorganization and structural–functional integration in TMD, especially within pain, emotion, and motor-control circuits; enables detailed mapping of cortico-subcortical and cerebello-cortical pathways relevant to nociceptive modulation; full-axis representation from cortex to medulla, Brainnetome should be combined with cortex, cerebellum and dedicated brainstem parcellations [3].

Schaefer (local–global; clarification, (Fig. 6b))—integrates local connectivity gradients with large-scale resting-state network structure, preserving the canonical Yeo 7/17-network architecture while refining intra-network subdivisions. Implemented at multiple resolutions (100–1000 ROIs), it enables systematic testing of spatial specificity and robustness of functional associations across parcellation scales [34]. Strengths: the dual-resolution flexibility of the atlas supports evaluation of network stability and reproducibility across granularities; by combining global network structure with local functional gradients, the parcellation improves anatomic plausibility and interpretability of network analyses; relevant for pain and motor research, enabling detailed characterization of reorganization within somatomotor, salience, and insular networks. Limitations: excludes subcortical, cerebellar, and brainstem regions and requires complementary atlases for full-axis analyses; higher spatial resolutions improve specificity but reduce ROI-level SNR and increase sensitivity to motion and smoothing artifacts; cross-resolution comparisons require normalization of network density and accounting for differences in ROI size. *Practical guidance for TMD studies* differentiating broad shifts between salience and default-mode systems from localized disruptions within somatomotor and insular networks; the chosen resolution should be explicitly reported to ensure reproducibility and comparability with prior TMD research [34].

Archer—sensorimotor area tract template (Fig. 6c)—probabilistic white-matter template depicting corticofugal tracts originating from six key sensorimotor cortical regions: primary motor cortex (M1), dorsal and ventral premotor cortices, supplementary motor area (SMA), pre-SMA, and primary somatosensory cortex (S1). Developed for diffusion MRI tractography, it characterizes descending sensorimotor pathways and enables assessment of projection-fiber integrity relevant to motor and pain modulation [44]. Strengths: provides anatomically and functionally defined probabilistic maps of major corticofugal bundles, supporting reproducible tract-based ROI analyses in DTI or tract-specific morphometry; highly suited to investigations of motor control, orofacial movement, and cortical modulation of jaw-related or trigeminal motor circuits; enables extraction of quantitative diffusion metrics (FA, MD, AD, RD) along descending tracts for between-group or longitudinal comparisons. Limitations: limited to white-matter tracts and does not include cortical gray-matter regions or subcortical, cerebellar, or brainstem structures; complementary atlases are required for whole-axis analyses; coverage is confined to corticofugal projections and does not include associative or limbic pathways. Practical guidance for TMD studies: is appropriate when examining the structural integrity or functional modulation of descending sensorimotor pathways relevant to TMD or orofacial pain, particularly in contexts involving mandibular movement, occlusal load, or chewing-related motor control; For integrative analyses across the full pain-modulatory axis, should be combined with another parcellation to establish coherent tract-to-region correspondence from the cortex through the cerebellum and into the medulla [44].

#### Integrative summary for TMD

distributed alterations across sensorimotor, salience/attention, limbic, and pain-modulatory circuits, making it unlikely that a single atlas can address all analytic requirements, the combined use of complementary parcellation schemes. HCP-MMP1.0 offers fine-grained, reproducible cortical localization; SUIT ensures accurate cerebellar normalization and ROI definition—particularly for Crus I/II, the vermis, and deep nuclei implicated in chronic pain and occlusal tasks; and specialized brainstem atlases enable precise assessment of infratentorial antinociceptive and arousal nuclei such as the PAG, RVM, and LC [3, 32–39]. Depending on study goals, Schaefer’s local–global model provides network-centric characterization of cortical remodeling in resting-state fMRI, while the Brainnetome atlas integrates structural and functional architecture across cortical, subcortical, and cerebellar regions (but lacks detailed brainstem parcellation) [3, 34, 35]. Anatomical atlases such as Desikan–Killiany/DKT and Destrieux support transparent reporting and comparability with legacy literature, balancing interpretability against granularity and SNR constraints [27–31]. Gordon and CAREN facilitate harmonization of network findings across functional atlas frameworks [41, 42]. Talairach remains useful for coordinate interpretation in older studies [40], and MICCAI 2012 provides a benchmark for validating segmentation workflows rather than serving as an ROI atlas [43]. For hypotheses targeting descending sensorimotor pathways, the Archer template adds tract-specific DTI sensitivity [44]. In practice, cross-atlas consistency is essential for capturing the full research paradigm. Using a common stereotaxic space (e.g., MNI)and statistical maps, standardized tissue segmentation, and sensitivity analyses across scales (e.g., multiple Schaefer resolutions) improves reproducibility and reporting clarity. Mechanistic insight is strengthened by aligning findings from task-based and resting-state fMRI and, when relevant, integrating DTI connectomes derived from cortical atlases (HCP/Schaefer) with tractography of sensorimotor pathways (Archer) in targeted analyses [3, 34, 44].

## Conclusions

Based on the analysis, the most informative approach for TMD is a combined scheme comprising HCP-MMP1.0 for detailed cortical mapping, SUIT for the cerebellum, and specialized brainstem atlases [3, 32–39]. Schaefer (local–global) and/or Brainnetome may be added as optional components for: network-level rs-fMRI analysis (Schaefer) or integrated structural-functional architecture with subcortical/cerebellar coverage (Brainnetome) [3, 34, 35]. Desikan–Killiany/DKT and Destrieux are anatomical references for comparison and interpretation [27–31], while Gordon/CAREN assist in aligning network findings across functional frameworks [41, 42]. Talairach remains a coordinate reference for localization [40], and MICCAI 2012 supports validation of segmentation pipelines [43]. When DTI hypotheses emphasize sensorimotor white matter, the Archer template is an appropriate reinforcement [44]. This multimodal combination increases mapping accuracy and meets modern requirements for the standardization of neuroimaging protocols in TMD research (Table 1).

**Table 1:** Comparing the table of the main human brain parcellation.

	Parceling scheme	Coverage	fMRI/DTI compatible	Research applications
1.	Desikan–Killiany (2006)	Crust (68 gyral regions). Subcortex/trunk as a single segment in the basic implementation. The cerebellum is not detailed.	It is widely used in fMRI (ROI analysis of large areas). Suitable for DTI connector (68 × 68 matrix). Limited detail can smooth out local effects.	Basic standard of anatomical parceling; has been used in numerous works on pain (stroke analysis, ACC, etc.) and cognitive control. Easy to interpret, but doesn’t take into account small function subdivisions.
2.	Destrieux (2009)	Cortex ( ≈ 148 regions: detailed convolutions + furrows). Subcortex/trunk—as in DK (rough).	Smaller ROIs for fMRI—higher localization accuracy, but smaller signal. For DTI gives more nodes; however, registration is more difficult.	It is used for finer anatomical analyzes (cingulate separation, TPJ, etc.). In pain/decisions, it is less common to explicitly mention, but can help distinguish between sub-regions of the cortex if hypotheses call for.
3.	HCP-MMP1.0 (2016)	Cortex (360 areas, multi-modal map). Very high detail. Does not cover the subcortex, cerebellum, trunk (they are segmented separately).	Optimized for fMRI on HCP Group Data: Exact Match to Function Fields. For DTI—Use is possible, but areas are very shallow (high-quality diffusion is required).	Modern network studies are used to accurately map the cortex (for example, different fields of the insula or SMA in pain studies). Provides reproducibility and standardization when comparing results between studies.
4.	Schaefer (2018)	Cortex (functional clusters; 100–1000 ROI options). Focused on 7 or 17 Yeo networks. Subcortex/cerebellum not included (can be added separately).	Ideal for rs-fMRI and network analysis (tuned to internal functional organization). In DTI—used for connectomes. Flexible choice of parceling scale for data resolution.	Common in functional connectivity research. In pain—to detect network restructuring (DMN, Somatomotor, Salience) during pain. In decision-making, to analyze the dynamics of networks of attention, control, etc.
5.	SUIT (2006/2009)	Cerebellum + trunk (high-section template, parceling of the cerebellum into 28 zones: I–X lobules, vermis, nuclei). The brainstem is represented in a pattern, but the detail is mostly on the cerebellum.	Specialized for cerebellum-fMRI: improves normalization and ROI analysis of the cerebellum. Compatible with DTI (better aligns the path of perforating fibers and legs). There are tools for projecting activations onto a flat map of the cerebellum.	It is necessary for the correct analysis of the role of the cerebellum. It is used in all modern fMRI studies where the focus is on the cerebellum (ataxias, cognitive functions of the cerebellum, the effect of pain on the cerebellum, etc.). Provides a standard nomenclature of particles for reporting results.
6.	Brainstem atlases	Brainstem. *FS:* 3 segments (midbrain, pons, medulla) + SCP. *Navigator:* 31 small nuclei (including medulla oblongata: Raphé, NTS, olives, etc.).	fMRI: Require high spatial accuracy (especially for Navigator). 7 T or special pulsation suppression methods are recommended. DTI: the presence of ROI nuclei helps to track pain tracts (e.g., spinoreticular pathway via RVM).	The newest sphere. In pain—it was possible to identify activity in PAG, RVM in analgesia; investigate the role of LC in chronic pain (due to its effects on arousal). In cognitive tasks—assessment of LC (norepinephrine effects on decisions), VTA (dopamine in reward).
7.	Talairach Atlas	Cortex, subcortex, cerebellum and trunk as single segments	It is used mainly for spatial mapping of coordinates; does not give separate ROIs for signal analysis	Historical coordinate standard; rarely used for modern ROI analysis
8.	Gordon et al. (2016)	Cora (333 functional patches)	Optimized for rs-fMRI (Correlation Clustering); limited anatomical match for DTI paths	Analysis of functional networks of rest; Study of Connectivity Change in Cognitive Tasks
9.	CAREN (2019)	Cora (5 large RSN consensus networks)	Created on the basis of several RSN atlases, suitable for fMRI; not designed for DTI	Standardization and comparison of the main functional networks
10.	MICCAI (2012)	Full brain (cortex, subcortex, cerebellum, trunk)	Used as a “golden” calibration for segmentations; is not a direct ROI atlas for fMRI/DTI	Training and validation of automatic segmentation algorithms
11.	Fan (2016)	Cortex (210 regions) and subcortex (36 nuclei)	Combines DTI tractography and fMRI connectivity; high-quality nodes for building structural and functional connectomes	Detailed study of the structural and functional architecture of the brain; analysis of pain networks and cognitive processes
12.	Schaefer (2018)	Cora (100–1000 ROI, Yeo network hierarchy)	Excellent for rs-fMRI network analysis; DTI connectors are possible, but anatomical correspondence is limited	Functional Connectivity Studies in Pain, Attention and Decision Making
13.	Archer (2017)	White matter of sensorimotor tracts (6 major tracts)	Specialized DTI Atlas for Sensorimotor Pathways; does not apply to fMRI	Analysis of the structural integrity and connectivity of sensorimotor tracts

## Discussion

Neuroimaging has to advance understanding of the neurobiological mechanisms underlying TMD and supports interdisciplinary treatment strategies [45]. Because chronic TMD involves multimodal alterations across cortical, cerebellar, and brainstem systems, choosing an optimal parcellation scheme remains challenging. Our analysis indicates that a combined framework using HCP-MMP1.0 for cortical mapping, SUIT for cerebellar normalization, and specialized brainstem atlases provides the most comprehensive and accurate coverage for studying TMD mechanisms [32–39]. HCP-MMP1.0 offers high areal resolution and multimodal derivation [32], enabling precise localization of cortical regions. SUIT is essential for examining cerebellar it preserves infratentorial morphology and supports detailed ROI analyses of lobules and deep nuclei [35]. Brainstem atlases, ranging from coarse structural maps to nucleus-level templates, enable investigation of antinociceptive and arousal systems. A further methodological requirement is evaluating concordance between parcellation schemes. Metrics such as the Rand Index, Adjusted Rand Index, and Adjusted Mutual Information provide quantitative assessments of similarity, correcting for chance agreements and accommodating differences in parcel number and size. Applying these measures improves reproducibility, supports harmonization across studies, and clarifies cross-atlas compatibility [46, 47].

In conclusion, the combined use of HCP-MMP1.0, SUIT, and specialized brainstem atlases currently provides the most informative multimodal framework for characterizing TMD-related neural alterations. Future research should validate this combined approach across cohorts, incorporate formal concordance metrics, and establish standardized reporting guidelines to strengthen clinical and research applications.

## Data Availability

No new data were generated or analyzed in this study. All data supporting this review are available from the cited literature.
